# Unsupervised Learning on Resistive Memory Array Based Spiking Neural Networks

**DOI:** 10.3389/fnins.2019.00812

**Published:** 2019-08-06

**Authors:** Yilong Guo, Huaqiang Wu, Bin Gao, He Qian

**Affiliations:** Institute of Microelectronics, Tsinghua University, Beijing, China

**Keywords:** unsupervised learning, spiking neural network (SNN), memristor, RRAM (resistive random access memories), 1T1R RRAM, STDP

## Abstract

Spiking Neural Networks (SNNs) offer great potential to promote both the performance and efficiency of real-world computing systems, considering the biological plausibility of SNNs. The emerging analog Resistive Random Access Memory (RRAM) devices have drawn increasing interest as potential neuromorphic hardware for implementing practical SNNs. In this article, we propose a novel training approach (called greedy training) for SNNs by diluting spike events on the temporal dimension with necessary controls on input encoding phase switching, endowing SNNs with the ability to cooperate with the inevitable conductance variations of RRAM devices. The SNNs could utilize Spike-Timing-Dependent Plasticity (STDP) as the unsupervised learning rule, and this plasticity has been observed on our one-transistor-one-resistor (1T1R) RRAM devices under voltage pulses with designed waveforms. We have also conducted handwritten digit recognition task simulations on MNIST dataset. The results show that the unsupervised SNNs trained by the proposed method could mitigate the requirement for the number of gradual levels of RRAM devices, and also have immunity to both cycle-to-cycle and device-to-device RRAM conductance variations. Unsupervised SNNs trained by the proposed methods could cooperate with real RRAM devices with non-ideal behaviors better, promising high feasibility of RRAM array based neuromorphic systems for online training.

## 1. Introduction

Spiking Neural Networks (SNNs) have been developed in the last decades as the third generation Artificial Neural Networks (ANNs) since SNNs behave more similarly to the natural neural systems, such as the human brain (Maass, 1997). The human brain is capable of complex recognition or reasoning tasks at relatively low power consumption and in a smaller volume, compared with those of training conventional ANN models of similar accuracy. The synaptic modification manners found in cultured hippocampal neurons introduced a great abstract model of the synaptic plasticity (Bi and Poo, 1998), namely the Spike-Timing-Dependent Plasticity (STDP). The STDP rule describes how the intermediate synapse changes its plasticity according to the spike timings of pre-neurons and post-neurons. The STDP rule could be armed as an unsupervised learning mechanism in SNNs, to implement more bio-like neural computing systems. However, SNN simulations require much more effort for preserving and utilizing the enormous amount of spatial-temporal information encoded in spike trains, thus are incredibly compute-intensive on conventional von Neumann computing systems. Some dedicated Very-Large-Scale Integration (VLSI) neuromorphic architectures have been proposed to enhance the neural simulation performance (Schemmel et al., 2010; Painkras et al., 2013; Qiao et al., 2015). VLSI technology allows intensive integration of neurons; however, the implementation of synapse arrays requires many transistors and intricate circuit designs, to emulate the learning and plasticity dynamics such as STDP. Recently, the analog Resistive Random Access Memory (RRAM) devices have become emerging neuromorphic hardware for artificial synapses, thanks to the controllability on their conductances and the ability of in-memory computing (Jo et al., 2010). The nanoscale fabricated RRAM devices can also be easily integrated as high-density crossbar arrays, which provide elegant solutions for the implementation of numerous synapses in neural systems. STDP allows the synapse to modulate its plasticity/strength according to the relative spike timing difference of the neurons connected by that synapse, and RRAM devices have been proved to be capable of providing various STDP characteristics (Jo et al., 2010; Yu et al., 2011b; Ambrogio et al., 2013, 2016; Wang et al., 2015; Pedretti et al., 2017; Wu and Saxena, 2017; Prezioso et al., 2018).

Typically, training neural network models *in-situ* on memristive devices could be challenging due to the device imperfectness and non-idealities, such as read noise, write noise, write nonlinearities, asymmetric SET/RESET switching behaviors and the limited gradual levels during programming (Agarwal et al., 2016; Chang et al., 2017; Wu et al., 2017). To accomplish recognition tasks such as learning handwritten digits (LeCun et al., 1998) with memristive neuromorphic hardware, Gokmen and Vlasov (2016) gave an estimate for the number of states that are required to be stored on a RRAM device as 600. While the reported state-of-art technologies allow the memristive devices to have 64 states (Park et al., 2016), up to over 200 states (Gao et al., 2015) continuously tuned by consecutive programming pulses, it is typically impossible to precisely control the conductance level using single shot programming (Kuzum et al., 2011; Yu et al., 2013; Eryilmaz et al., 2016). For neural networks trained with supervision, such as backpropagation (LeCun et al., 1989), the conductance of memristive devices can be fine-tuned to the desired value during the training process, using write-verification scheme (Guan et al., 2012; Yao et al., 2017), which introduces operation overheads to modulate the device conductance more precisely.

However, when it comes to unsupervised neural networks such as SNNs trained with STDP, write-verification scheme is not compatible with unsupervised learning since there is no error propagating backward and the weights should be self-adaptive to the input stimulus and output responses (STDP). Therefore, the switching behavior under consecutive programming pulses of RRAM devices is essential for implementing unsupervised learning algorithms. The dynamic range and minimum achievable mean conductance change will limit the learning rate of training algorithms (Gokmen and Vlasov, 2016). The learning rates for typical SNN training algorithms are set at the magnitude order around 10^−4^ ~ 10^−2^ (Masquelier and Thorpe, 2007; Querlioz et al., 2013; Panda et al., 2018), which implies at least 100~ 1, 000 intermediate states are needed for RRAM devices to implement such learning rules without compromise. So far, memristive device technologies could provide with devices of <100 multi-level states (Kuzum et al., 2013), which limits the complexity of RRAM-based SNNs. Several SNNs of simple structures have been simulated or demonstrated basing on memristive devices (Wang et al., 2015; Pedretti et al., 2017), accomplishing recognition tasks such as 4 × 4 binary patterns with one post-neuron (Pedretti et al., 2017), 3 × 3 binary patterns with two competitive post-neurons (Pedretti et al., 2017) and one single 8 × 8 pattern with eight pre-neurons and eight post-neurons (Wang et al., 2015). The abrupt switching behavior of RRAM devices limits the complexity of recognition tasks accomplished by unsupervised SNNs. Boybat et al. (2018) have proposed an architecture to wrap several Phase Change Memory (PCM) devices as one single synapse, to reduce the smallest achievable mean conductance change, therefore improving the effective conductance change granularity. This N-in-1 (N PCMs serving as one single synapse) architecture requires additional arbitration control circuit to manage N PCMs for each synapse. Their unsupervised SNN simulation with device model achieves remarkable performance by using 9-in-1 architecture (9 PCMs as one synapse), reaching testing accuracy over 70% on MNIST dataset with a single-layer (no hidden layer) SNN of 50 post-neurons, which is close to the float-precision baseline 77.2% (Boybat et al., 2018).

In this work, we propose a novel scheme for training unsupervised SNNs, with pattern/background phases and greedy training, to cooperate with realistic RRAM characteristics. The pattern/background phases and greedy training methods allow input pattern spike trains to have much lower frequencies and still guarantee the synapses to learn correct patterns and forget irrelevant information as well. Lower firing rate of neurons in SNNs will lead to fewer times of conductance changes for RRAM devices. We conduct simulations of unsupervised SNNs for the recognition of the handwritten digits from MNIST dataset, as well as the SNNs with different levels of RRAM cycle-to-cycle and device-to-device variations. The testing accuracy for 10,000 test images from MNIST dataset reaches around 75% after single-epoch unsupervised learning on 60,000 training images, with 30% cycle-to-cycle and device-to-device write variation, together with 10% cycle-to-cycle, and device-to-device dynamic range variation. The SNNs trained with proposed training methods show excellent performance even with large learning rates, which indicates that the requirement for the number of levels of RRAM devices could be reduced, and the abrupt switching, asymmetric switching could also be tolerated well. The unsupervised SNNs trained with proposed training methods show high feasibility of RRAM array based neuromorphic systems for online training.

In this article, the material details of our 1T1R device will first be introduced in section 2.1. Then the STDP architecture on 1T1R array and the unsupervised SNN architecture will be explained in sections 2.2, 2.3 respectively. The STDP characteristic of 1T1R devices measured from experiment is shown in section 3.1. The pattern/background phases and greedy training methods are described in sections 3.2.1, 3.2.2. The inference technique is also included in section 3.2.3. And classification results on digit recognition are shown in sections 3.2.4, 3.2.5. In section 4, more types of RRAM non-idealities are discussed, such as endurance, failure rate and asymmetric switching behavior. Section 5 highlights the main contributions of this work.

## 2. Materials and Methods

### 2.1. 1T1R Device

**Figure 1:**
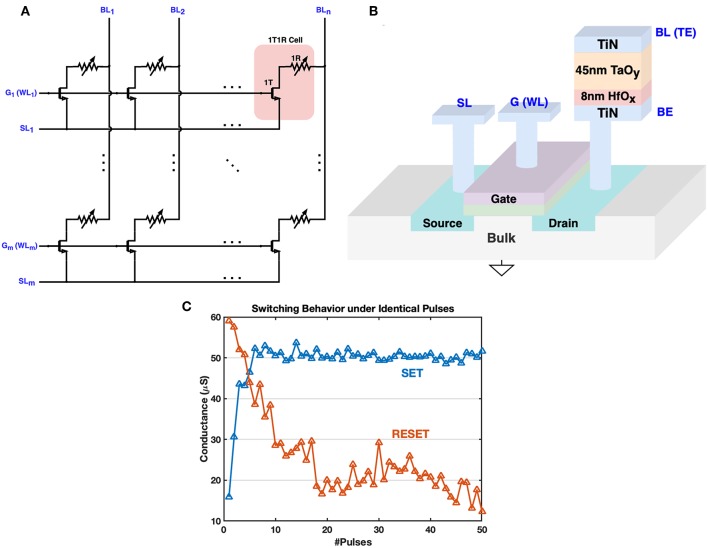
The architecture of 1T1R crossbar array and 3D fabrication illustration. **(A)** The 1T1R crossbar array layout. The transistor gates and transistor sources of 1T1R cells in the same row are connected to the G (WL) bus and SL bus respectively. The RRAM top electrodes (TE) of 1T1R cells in the same column are gathered onto the BL bus. **(B)** The 3D fabrication structure schematic of the 1T1R cell. The bottom electrode (BE) of each RRAM device is connected to the transistor drain node., and the top electrode (TE) is wired with the BL bus. When the transistor gate is open by the high voltage on the WL bus, a positive voltage across BL and SL will help to strengthen conductive filaments in the HfO_x_/TaO_y_ layer, increasing the RRAM conductance, which is known as the SET operation. FORM operation is similar but with a higher positive voltage across BL and SL, to form the main conductive filaments in the TaO_y_ layer for the first time. RESET requires a reverse operation voltage that tries to cut off the filaments formed in the HfO_x_ layer, thus decreasing the RRAM conductance. **(C)** Typical switching behavior of our 1T1R device under consecutive identical operation pulses (width = 50 ns) during SET/RESET. *V*_BL_ = 1.5V, *V*_G_ = 2.0V, *V*_SL_ = 0 for SET, and *V*_SL_ = 1.4V, *V*_G_ = 4.0V, *V*_BL_ = 0 for RESET. Abrupt switching is more readily observed during SET.

The one-transistor-one-resistor (1T1R) structure is used to fabricate the RRAM crossbar array, as illustrated in Figure 1. Each RRAM device consists of a TiN/TaO_y_/HfO_x_/TiN stack. The transistor inside the 1T1R cell plays an important role to overcome the shortcomings of the conventional 2-terminal 1R or one-selector-one-resistor (1S1R) crossbar array, such as sneak current path and programming disturbance (Yao et al., 2015). Furthermore, the gate node offers more control over the whole 1T1R cell since the current through the device can be complied during the SET processes. The control on gate voltage allows more immunity to the switching voltage magnitude and achieves better uniformity (Liu et al., 2014).

Each 1T1R cell has three main terminals: transistor gate, top electrode and transistor source, and they are connected to the word-line (WL, also noted as G), bit-line (BL), and source-line (SL) respectively in the array layout. Typical switching behavior during SET and RESET is shown in Figure 1C, where abrupt switching during SET is observed since the generation of each oxygen vacancy during the SET process can increase the local electric field/temperature and accelerate the generation of other vacancies, analogous to avalanche breakdown (Yao et al., 2017). Gate voltage pulses are usually different during SET and RESET processes: lower gate voltage is applied during SET to limit the set current, while RESET process requires a higher gate voltage to supply adequate reset current (Wu et al., 2011; Yao et al., 2017). Furthermore, we can notice that the switching behaviors of SET and RESET are asymmetric, which is one of the major bottlenecks that limit the performance of memristive-based neural computing system (Kuzum et al., 2013). Fortunately, this asymmetric behavior could be partly compensated by tuning device-independent parameters of proposed training methods. In the next section, we introduce an architecture for 1T1R crossbar array to implement the biological plausible STDP feature of synapses. This schematic is a general design which can be configured for different 1T1R devices that require different operating voltages.

### 2.2. STDP Implementation on 1T1R Array

**Figure 2:**
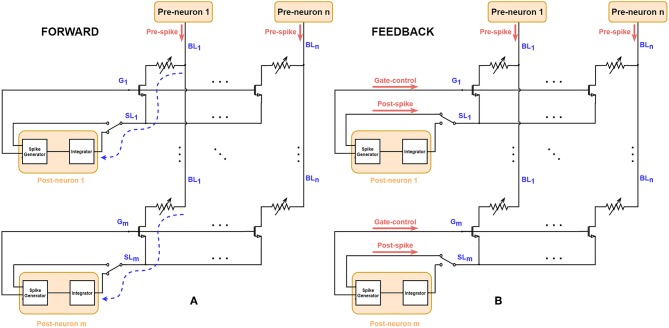
Schematic for FORWARD/FEEDBACK modes on 1T1R RRAM array. Each Leaky-Integrate-and-Fire neuron (namely post-neuron) is connected to the SL and G nodes and each Poisson neuron (pre-neuron) is connected to the BL. **(A)** In FORWARD mode, the current stimulated by input pre-spikes can flow through the 1T1R cell and finally arrives at the integrator module of post-neurons (marked as dashed blue curve), where the input information encoded in pre-spikes is conveyed to the post-neurons. **(B)** When the post-neurons generate output signals, i.e., post-spikes and gate-controls, the circuit changes to the FEEDBACK mode via the control of the two-state switch at SLs. The conductance of RRAM devices could be programmed since the Gate is enabled and there is a voltage across the RRAM devices because of the simultaneous presence of pre-spikes and post-spikes.

Figure 2 shows the schematic to implement STDP characteristics basing on the 1T1R array, where each 1T1R cell acts as one electrical synapse. The pre-neuron layer is connected to the synapse array via *n* BLs, and the post-neuron layer is connected to *m* SLs, representing the fully-connected structure of two layers in topology. In the forward mode, when the pre-spike voltage signal is applied on the BL, corresponding current flows through 1T1R cell and adds up with the current of other cells in the same row at the SL node. This current stimulates the post-neuron (leaky-integrate-and-fire neuron) to integrate and modify the membrane voltage. Once the membrane voltage of the post-neuron reaches a certain threshold, the spike generator module will generate two synchronized spike signals: post-spike and gate-control. In the feedback mode, the gate line is controlled by a certain pulse generated by post-neuron, for the RRAM SET/RESET processes. The voltage across the given memristor (i, j) is determined by the voltage difference of BL_j_ and SL_i_. So the overlapped waveform of pre-spikes and post-spikes with some time window will determine the behaviors of 1T1R cells during the feedback process. This design provides a flow paradigm with two communication phases and allows parallel modulation on crossbar states utilizing the overlapped spiking events naturally. Thanks to the crossbar architecture which binds all Gate nodes and Source nodes of all devices in one row, the temporal all-to-all spike-interaction of STDP could be implemented easily (Morrison et al., 2008). Similar structures on STDP implementation have been proposed for 1R (RRAM without any transistor, also known as 0T1R) devices (Yu et al., 2011b; Wu and Saxena, 2017; Prezioso et al., 2018), while for 1T1R devices, additional control on Gate nodes is required.

**Figure 3:**
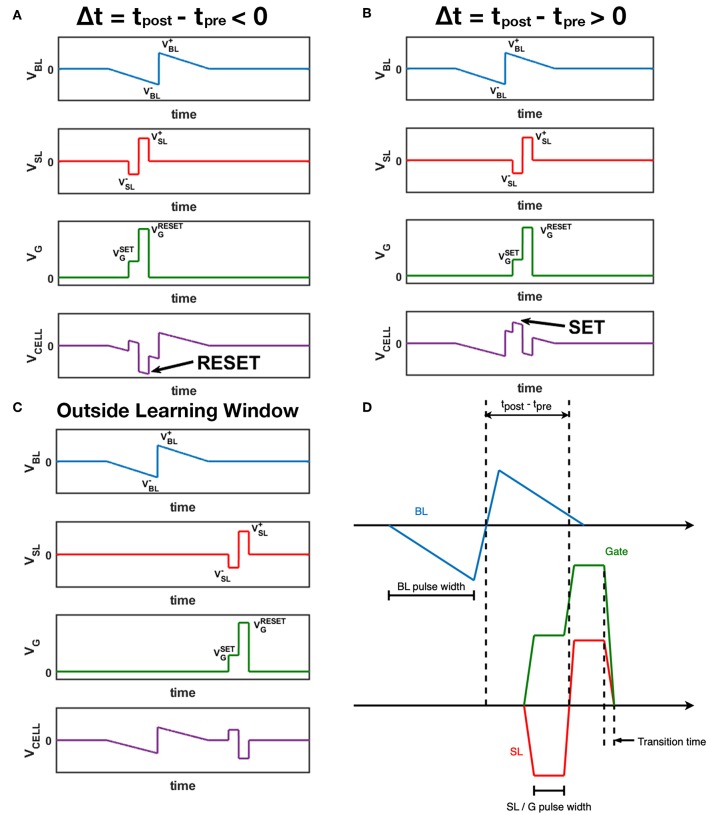
Waveform design for BL (pre-spikes), SL (post-spikes) and Gate. The voltage across the 1T1R cell is also displayed as V_CELL_, which equals to V_BL_ – V_SL_. **(A)** A post-spike that fires right before the pre-spike event. **(B)** A post-spike that fires right after the pre-spike event. **(C)** A post-spike that fires without overlapping of the pre-spike event. **(D)** Time parameters of three channels. The transition time of all channels are the same, and SL pulses and Gate pulses have the same synchronized width.

Figure 3 shows the abstract waveform design for the STDP architecture mentioned above. According to the STDP rule observed in natural neural system (Bi and Poo, 1998), when the post-spike fires slightly before the pre-spike, the synapse should be depressed, and for the RRAM device, the conductance should decrease. As illustrated in Figure 3A, the positive part of post-spike pulse overlaps with the negative part of the pre-spike pulse, causing a larger negative voltage across the 1T1R cell, which in fact is a RESET operation given the appropriate gate voltage, leading the synapse conductance to a lower value. Similarly in Figure 3B, when the post-spike follows the pre-spike closely, the voltage across the cell is a large positive value which can SET the device into a higher conductance state. Figure 3C shows the situation that the pre-spike does not overlap with the post-spike, and no learning mechanism is triggered. The peak positive voltage values of BL and SL are annotated as V_BL_^+^ and V_SL_^+^, and V_BL_^–^, V_SL_^–^ for the negative parts. V_G_^SET^ and V_G_^RESET^ represents the appropriate gate voltage during SET and RESET respectively. Analytically, magnitude of the voltage across the cell varies from |V_SL_^–^| to V_BL_^+^ + |V_SL_^–^| during SET, from V_SL_^+^ to V_SL_^+^ + |V_BL_^–^| during RESET. These pulse shaping parameters (including V_G_^SET^, V_G_^RESET^ and pulse width) can be configured with flexibility to meet the control requirements of different 1T1R devices and for desired synaptic characteristics (Figure 3D). The STDP characteristic shown by our 1T1R devices under this scheme design is experimentally measured in section 3.1.

### 2.3. Unsupervised SNN Architecture

**Figure 4:**
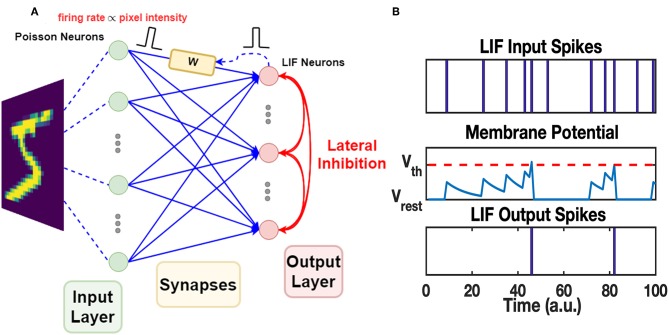
The architecture of SNN and mechanism of LIF neuron. **(A)** The two-layer SNN architecture. The input layer is responsible for converting input images into spike trains. Poisson neurons are used in this layer. The spikes generated by the input layer are transmitted to the synapses in the middle, fully connecting the input neurons and the output neurons. The synapses modulate the received spikes (defined as pre-spikes) by their weights and pass the spikes to the output layer. LIF neurons in the output layer process the spikes and generate output spikes properly. The mechanism of LIF neuron is explained in **(B)**. The output spikes (defined as post-spikes) are passed back to the corresponding synapses and tune the synapse weights via STDP rule. Additionally, output spikes are broadcasted among output neurons through the lateral inhibition paths, allowing competition during learning. **(B)** LIF neuron firing mechanism. The LIF neuron has an internal state, i.e., membrane potential. It integrates on the presence of received input spikes and decays exponentially with a time constant τ_mem_. Once the membrane potential reaches a certain threshold V_th_, it fires a spike at the output port and the membrane potential is reset to the resting potential V_rest_. The fired LIF neuron itself then enters into a short refractory period, when its membrane potential holds at V_rest_ and does not respond to any recent input spikes.

The work uses a Spiking Neural Network which consists of two layers of neurons, as shown in Figure 4A. The neurons in the input layer are Poisson neurons which produce spike trains whose firing rate is proportional to the associated pixel intensity (Diehl and Cook, 2015; Boybat et al., 2018). For one gray-scale image stimulus, the 2-dimensional image will be flattened into a 1D vector, and each pixel is mapped to one input Poisson neuron. The Poisson neurons are fully connected to a layer of Leaky-Integrate-and-Fire (LIF) neurons, serving as the output layer. The mechanism of one LIF neuron is explained in Figure 4B. In the forward mode, each synapse in the middle conveys the spike signals of the certain input neuron to the output neuron via its strength, defined as *W*. In the feedback (backward) mode, the strength of the synapse is modified according to the pre-spike and post-spike timings. The STDP variant rule, which changes weight with soft bound is used (Kistler and Hemmen, 2000), as shown in Equation 1. The relative weight changes Δ*W*/*W* of soft bound STDP model vary with different *W* states (see Equation 2). In general, when applying the same SET operation on RRAM devices in HRS, the consequent relative conductance change is often larger than that of devices in lower resistance states, and similarly for the RESET operation. This nonlinear manner of RRAM devices matches the synapse strength modulation modeled by soft bound STDP. The STDP model with soft bound fits better with the experimental behavior of the 1T1R device under the STDP circuit architecture and waveform design mentioned above, as explained in section 3.1. Δ*t* is defined as *t*_post_−*t*_pre_, where *t*_post_ and *t*_pre_ represent the spike timings of the post-neuron and pre-neuron respectively. While the classical STDP model which expects the relative weight changes to be irrelevant with original weight states (see Equation 3) does not match the typical nonlinear behaviors of RRAM devices.

(1) $$\Delta W=\left\{ \begin{array}{l} A_{+}(W_{\text{max}}-W)\text{ exp }\left(-\frac{\Delta t}{\tau_{+}}\right),\text{ if }\Delta t>0\\ -A_{-}(W-W_{\text{min}})\text{ exp }\left(-\frac{\left|\Delta t\right|}{\tau_{-}}\right),\text{ if }\Delta t<0 \end{array}\right.$$

(2) $$\frac{\Delta W}{W}=\left\{ \begin{array}{l} A_{+}\left(\frac{W_{\text{max}}}{W}-1\right)\text{ exp }\left(-\frac{\Delta t}{\tau_{+}}\right),\text{ if }\Delta t>0\\ -A_{-}\left(1-\frac{W_{\text{min}}}{W}\right)\text{ exp }\left(-\frac{\left|\Delta t\right|}{\tau_{-}}\right),\text{ if }\Delta t<0 \end{array}\right.$$

(3) $$\frac{\Delta W}{W}=\left\{ \begin{array}{l} A_{+}\text{ exp }\left(-\frac{\Delta t}{\tau_{+}}\right),\text{ if }\Delta t>0\\ -A_{-}\text{ exp }\left(-\frac{\left|\Delta t\right|}{\tau_{-}}\right),\text{ if }\Delta t<0 \end{array}\right.$$

Since the synapse strength is modulated by STDP rule in an unsupervised manner, competition mechanism is required for the post-neurons to learn discriminated patterns (Masquelier et al., 2009; Carlson et al., 2013; Diehl and Cook, 2015; Panda et al., 2018). Lateral inhibitory paths are added to the output neurons in Winner-Take-All (WTA) fashion: once a LIF neuron fires at *t*_post_, membrane voltage of all neurons in the output layer will be reset to the resting voltage, and the spiking neuron itself goes into a refractory period as illustrated in Figure 4B. All other neurons need to re-accumulate their membrane voltage from resting voltage, and the spiked one will be held at resting potential during refractory, allowing LIF neurons to compete with each other for the firing opportunity. Furthermore, the homeostasis mechanism is also introduced among LIF neurons. The membrane threshold of each LIF neuron is adapted according to its recent spiking activity: threshold of the LIF neuron with more recent firing events will increase to lower its firing opportunity during the next several stimuli, and vice versa.

The training methods, namely pattern/background phases and greedy training, which allow the SNN to cooperate with large conductance change step shown by real RRAM devices will be introduced later in section 3.2, where the performance on the MNIST recognition tasks is also discussed.

## 3. Results

### 3.1. STDP Characteristic of 1T1R Device

**Figure 5:**
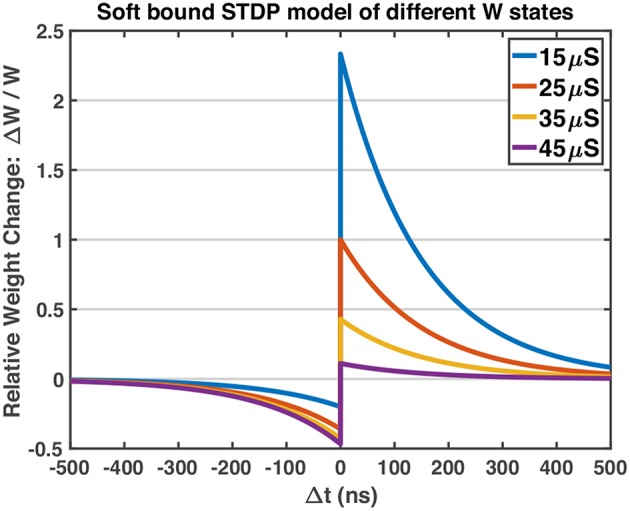
The STDP model curves of different *W* states. The potentiation of lower conductance states is stronger than that of higher conductance states, and vice versa for the depression process. Model parameters: *A*_+_ = 1.0, *A*_−_ = 0.6, τ_+_ = τ_−_ = 150 ns, *W*_max_ = 50 μS, *W*_min_ = 10 μS. *A*_−_ is set to be smaller than *A*_+_, which fits the experimental behavior of RRAM devices in Figure 6.

**Figure 6:**
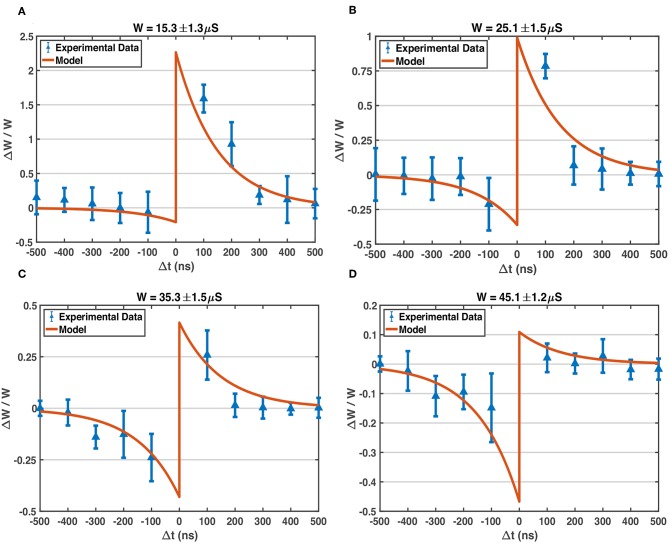
Experimentally measured STDP characteristic of our 1T1R devices, compared with the model. The waveform parameters of BL, SL, G pulses applied on the devices: V_BL_^+^ = 0.6 V, V_BL_^–^ = –1.0 V, V_SL_^+^ = 1.3 V, V_SL_^–^ = –1.0 V, V_G_^RESET^ = 4.0 V, V_G_^SET^ = 1.0 V, pulse width of V_BL_ = 500 ns, pulse width of V_SL_ and V_G_ = 50 ns, all transition time = 20 ns. The model parameters used here to compare with experimental data are the same as those listed in Figure 5: *A*_+_ = 1.0, *A*_−_ = 0.6, τ_+_ = τ_−_ = 150ns, *W*_max_ = 50 μS, *W*_min_ = 10 μS. **(A)** The experimentally measured data on 1T1R devices (blue points with errorbar) via Keithley 4200A-SCS equipment, and model-predicted STDP curve, around *W* state of 15.3 μS. Each plotted experimental data point is the average relative conductance change of over 100 trials, and the standard deviation is shown by the corresponding errorbar. In each trial, the device under test is fine-tuned to the target conductance state first, and then pulses are applied to device terminals for once, finally the conductance change is measured. **(B)** Measured STDP and modeled STDP around *W* state of 25.1 μS. **(C)** Measured STDP and modeled STDP around *W* state of 35.3 μS. **(D)** Measured STDP and modeled STDP around *W* state of 45.1 μS.

As mentioned above, the soft bound STDP (Equation 1) models different relative weight changes of different weight states (Equation 2), and the STDP model curves of different *W* states are plotted in Figure 5. The programming pulses of designed waveforms (Figure 3) are applied to 1T1R devices repeatedly with different initial states using Keithley 4200A-SCS, and the conductance changes of devices are measured. Figure 6 shows the obtained experimental data provided with detailed operation information, indicating that the designed pulse waveforms can modulate the 1T1R devices' conductance similar to the synapse behavior modeled by soft bound STDP.

The *A*_+_, *A*_−_ parameters in Equation 1 could be regarded as the learning rate of the STDP model. For our devices, the typical fitted value of *A* is larger than 0.5, up to 1.0, which indicates strong potentiation and depression processes (abrupt switching shown in Figure 1C) of the RRAM devices. The advance in material and structure of RRAM devices will lead to more ideal behaviors, such as gradual conductance switching, linear switching and more stable intermediate conductance states, which would allow us to model the learning mechanism with smaller learning rates. In typical SNN training algorithms, the learning rates are set at the magnitude order around 10^−4^~10^−2^ (Masquelier and Thorpe, 2007; Querlioz et al., 2013; Panda et al., 2018), which would face immense difficulties applying on current general RRAM devices directly without other circuit aids. To cooperate with the non-ideal abrupt switching on RRAM conductances, we propose a novel training workflow for SNNs, named as pattern/background phases and greedy training methods (see sections 3.2.1, 3.2.2), which show immunity to large conductance changes as well as the device variations.

### 3.2. SNN Performance on MNIST

#### 3.2.1. Encoding Input: Pattern/Background Phases

MNIST handwritten digits dataset is used as the application proof of SNNs trained with proposed methods. The dataset consists of 60,000 28-by-28 gray-scale images for training, and other 10,000 unseen images of the same size for testing phase^1^. Each Poisson neuron in the input layer is responsible for converting one pixel of the input image into a temporal spike train. The generated spike events are subject to Poisson distribution and firing rate of the Poisson neuron is proportional to the corresponding pixel's intensity (Diehl and Cook, 2015). At each simulation timestep, independent Bernoulli trials are conducted to determine whether to fire a spike event (Boybat et al., 2018). Additionally, the original gray-scale images from MNIST dataset are normalized by their total pixel intensity respectively before stimulating the Poisson neurons.

For each input image, the input encoding scheme includes a pattern phase and a background phase. During the pattern phase, the original image is fed to the input neurons; therefore, the pattern pixel (of higher intensity) channels are likely to have more spikes generated. During the following background phase, the complementary of the original image is used to stimulate the input layer for another period. The Poisson neurons connected to the background pixels (of lower intensity in the original image) spike more frequently in the background phase, to depress the irrelevant synapses which are mapped to the background pixels.

#### 3.2.2. Greedy Training

**Figure 7:**
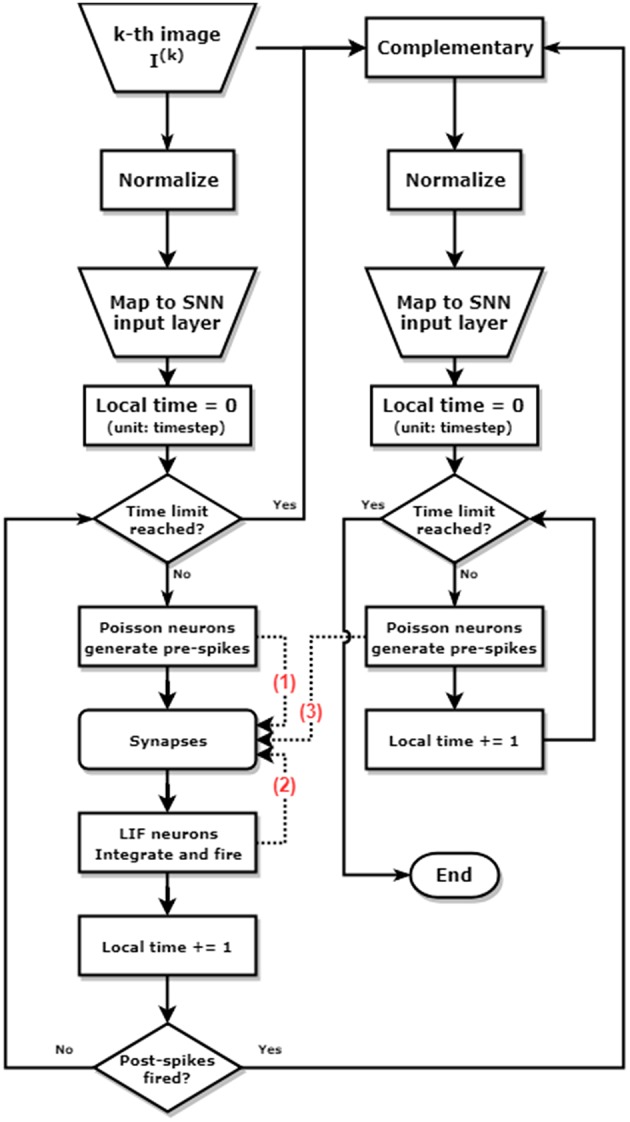
Block diagram of pattern/background phases and greedy training methods for learning one single image. The left and the right column represent the pattern phase and background phase respectively. The dashed lines annotated with number show the different timing when spikes arrive at the synapses. The pre-spikes in the pattern phase (1) arrive first, then the post-spikes from LIF neurons (2), and finally the pre-spikes generated by the Poisson neurons in the background phase (3). This spikes arriving sequence allows potentiation during pattern phases [synapses modulated by (1) and (2)], and depression during background phases [synapses modulated by (2) and (3)].

**Figure 8:**
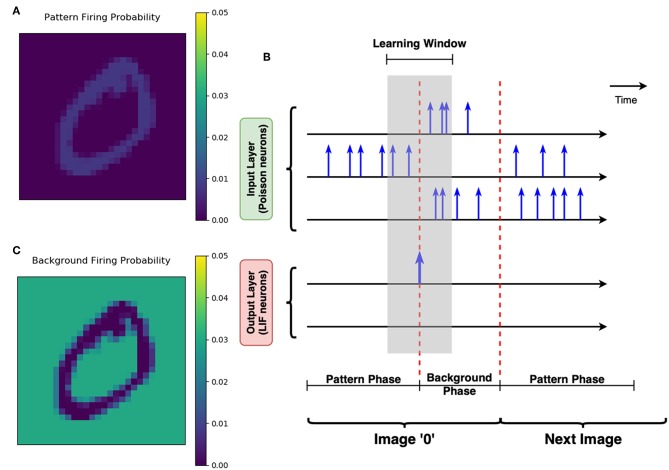
Greedy training and pattern/background phases. **(A)** The schematic of greedy training. Each row with the time axis represents the spiking activity of one neuron over time. Spikes are marked out by the blue vertical arrows. There are two phases for each image stimuli. During the pattern phase, the input neuron corresponding to the pattern pixels are more likely to fire spikes. The pattern phase continues until any of the LIF neurons at the output layer spikes. The LIF neuron's spike switches the input layer into the background phase immediately, allowing the background pixels to strongly stimulate the Poisson neurons. Therefore, the learning window shown by the gray shaded area could help the synapses learn the pattern and forget the irrelevant background efficiently. **(B)** The firing probability map of Poisson neurons in one single time step. This subfigure shows that the neurons corresponding to the “0” pattern have a higher firing probability during the pattern phase. **(C)** The firing probability of all pixels during the background phase. The Poisson neurons associated with background pixels are strongly stimulated during the background phase, to enable efficient forgetting of the network.

The simulation is conducted at a time step of 50 ns, to match the time scale of the waveform configurations mentioned in Figure 6. The routine of the training process can be described as follows and shown as the block diagram in Figure 7:

1. Get the *k*-th image *I*^(*k*)^ from MNIST training set.
2. Normalize *I*^(*k*)^ by its total pixel intensity. Let $I_{i}^{\left(k\right)}$ be the intensity of the *i*-th pixel (*i* = 1, 2, ⋯ , 784), then ${Ĩ}_{i}^{\left(k\right)}\leftarrow I_{i}^{\left(k\right)}/\underset{i}{\sum }I_{i}^{\left(k\right)}$.
3. Pattern phase: The normalized ${Ĩ}_{i}^{\left(k\right)}$ is mapped to the *i*-th Poisson neuron *P*_*i*_ in the input layer. For *P*_*i*_, the probability to fire a spike at a given time *t* equals to $f_{\text{pattern}}\times {Ĩ}_{i}^{\left(k\right)}$, where *f*_pattern_ is a factor to control the overall activity of the input layer. Note that $\underset{i}{\sum }f_{\text{pattern}}{Ĩ}_{i}^{\left(k\right)}=f_{\text{pattern}}$, which represents the average number of total spiking events in the input layer at a single time step, as shown in Figure 8A. In this work, *f*_pattern_ = 1 is used for all simulations, so that the average firing rate of one Poisson neuron is 1/(50ns × 784)≈25.5kHz.
4. The duration of the pattern phase is variable, with a maximum of 10 μs (200 steps). Ĩ^(*k*)^ is persisted to stimulate input layer until one post-neuron finally reaches its membrane threshold and fires a post-spike. Then pattern phase is switched to background phase immediately. The input layer expects to activate only one post-neuron during the pattern phase, this is so-called “greedy” training (Figure 8B).
5. Background phase: The complementary version of *I*^(*k*)^ is defined as Ī^(*k*)^ = 255−*I*^(*k*)^. Normalization is also conducted to the complementary image, such that normalized ${\tilde{Ī}}_{i}^{\left(k\right)}\leftarrow {Ī}_{i}^{\left(k\right)}/\underset{i}{\sum }{Ī}_{i}^{\left(k\right)}$. Similarly, the normalized complementary image stimulates the input layer by a factor *f*_background_ = 7, resulting in an average firing rate of one Poisson neuron at around 128kHz, as illustrated in Figure 8C. The background phase has a constant duration of 500 ns (10 steps).
6. Training iteration process of image *I*^(*k*)^ is completed. Get the (*k*+1)-th image from MNIST training set. Repeat from step 2 to step 6.

For LIF neurons in the output layer, the membrane time constant τ_mem_ = 10 μs. Resting membrane potential *V*_rest_ = 0V, and initial firing threshold is set as *V*_th_ = 0.4V. The refractory period is disabled for simplicity. Winner-Take-All rule is used for lateral inhibition, that is, only one LIF neuron in the same layer is allowed to fire in any single time step (Masquelier et al., 2009). Once some neuron fires a spike, membrane potentials of all neurons in that layer are reset to *V*_rest_. If more than one neuron's membrane potential increases over the firing threshold in one simulation time step, the one that exceeds its threshold the most is fired. The threshold of each LIF neuron is adapted through homeostasis: it increases by 0.1 × (*A*−*T*) at every new image input, where *A* represents the average number of spikes per time step for recent 1,000 images' training iterations, and *T* is the target number of spikes per time step (Boybat et al., 2018).

For synapses which fully connect the input and output layers, the soft bound model defined by Equation 1 is used. The parameters fitted with device experimental behaviors are used: *A*_+_ = 1.0, *A*_−_ = 0.6, τ_+_ = τ_−_ = 150ns, *W*_max_ = 50 μS, *W*_min_ = 10 μS. Initial synapse weights are uniformly distributed in [*W*_min_, *W*_max_].

#### 3.2.3. Inference Process

After iterating over all training images for one time, the network will be set to static inference mode. The synapse weights and membrane thresholds of LIF neurons will remain unchanged during the inference process. The lateral inhibition mechanism is still enabled to allow competition among output neurons, and the greedy manner is also kept, therefore once some post-neuron fires a spike for the input stimulus, the inference for this input is completed. The training images are applied to the network once again, and each image is persisted to stimulate the network until some post-neuron fires. The fired neuron index and firing time are recorded. Each image with label gives the fired neuron a confidence score as $\frac{1}{\text{firing time}}$ for the corresponding label, which indicates that the earlier the output neuron fires, the more confident the neuron is. The scores are summed up for each neuron and label after the stimulation of all training images, and all the LIF neurons are marked with the label with the highest summed confidence score. Then for any input image, once some post-neuron fires, the label corresponded with that neuron is recorded as the predicted label, which could be compared to the truth label. Therefore the recognition accuracy could be evaluated.

#### 3.2.4. Performance Without Variations

**Figure 9:**
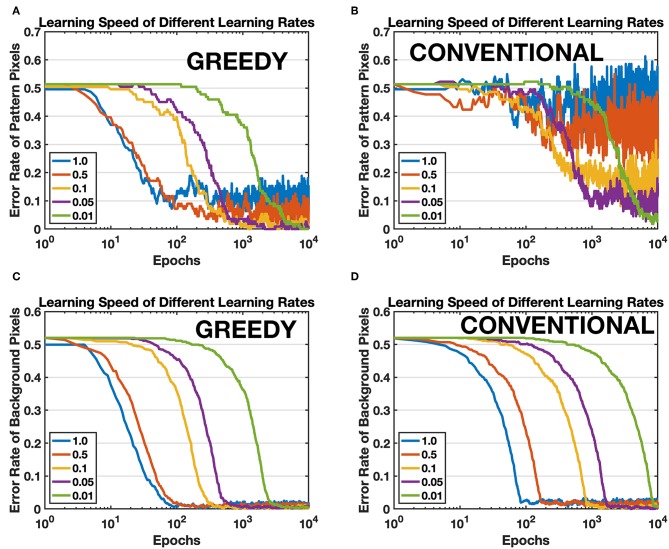
Comparison between the proposed training method (GREEDY) and conventional training method (CONVENTIONAL) on learning one single pattern. **(A)** Error rate of pattern pixels versus training epochs of greedy training. **(B)** Error rate of pattern pixels versus training epochs of conventional training. The convergence of conventional training with large learning rates is much worse than that of greedy training. **(C)** Error rate of background pixels versus training epochs of greedy training. **(D)** Error rate of background pixels versus training epochs of conventional training.

First of all, a single pattern learning task is conducted by using proposed greedy training method (pattern/background phases technique is always included for greedy training in this article unless explicitly pointed out) and conventional training method respectively. The conventional training method is armed with self-decaying techniques to forget irrelevant information more rapidly (Panda et al., 2018). The target pattern is the first image of MNIST, a handwritten digit “5.” The network consists of 784 input neurons and one single output neuron. All parameters for both training methods keep the same, except for some unique method-specific parameters such as background firing rate for greedy training and decay factor for conventional training. The efficacy of synapses is compared with the target pattern after learning since there is no supervision and competition among output neurons, and an ideal learning method should be able to learn all the details of the pattern. Therefore, the error rates of pattern pixels and background pixels are calculated to evaluate the learning accuracy, as shown in Figure 9. The network is trained by both methods under different learning rates, and Figures 9A,B show that the proposed greedy training has a better convergence especially when the learning rate is larger, and the speed for both methods is comparable (see green curves). Moreover, greedy training is also able to depress the irrelevant background synapses with the same speed as the self-decaying mechanism (Panda et al., 2018), shown in Figures 9C,D. The proposed training method lowers the requirement for the device characteristics, at least in terms of the minimal achievable conductance change.

**Figure 10:**
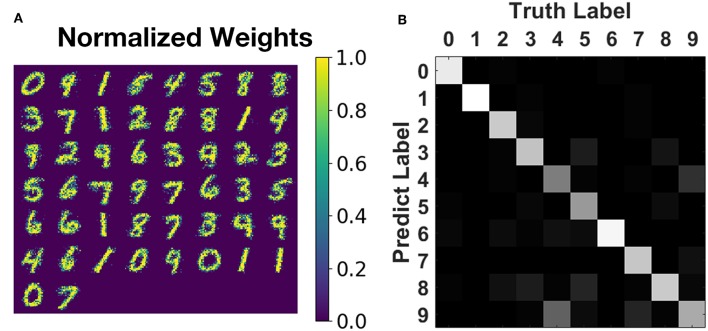
Training result on MNIST recognition. **(A)** The normalized weight map corresponding to 50 post-neurons. Most patterns of 10 digits are impressively learned without any supervision. **(B)** Testing accuracy on MNIST testing set of 10,000 unseen images during training. The overall testing accuracy is around 76.8%, and most of the categories could be classified with acceptable accuracy.

We have also trained an SNN with 784 input neurons and 50 output neurons to learn and recognize the full MNIST dataset. The network is of the same structure as the one in Boybat et al. (2018) but is trained by the proposed greedy method. The parameter values are set to be device compatible as mentioned in the caption of Figure 6 and section 3.2.2: timestep = 50 ns and *A*_+_ = 1.0, *A*_−_ = 0.6, τ_+_ = τ_−_ = 150 ns, *W*_max_ = 50 μS, *W*_min_ = 10 μS, *f*_pattern_ = 1, *f*_background_ = 7. The learning window width for STDP rule is set as four timesteps to reduce the number of update operations. The pattern phase of each training image is persisted for 200 time steps at most (since the greedy algorithm may finish the learning of this image ahead of time), and the background phase lasts for ten timesteps. Sixty thousand images from the MNIST training set are fed to the network sequentially (dataset order is not changed), and each image is learned only once. The training process finishes after around 9.6 million timesteps, which indicates that the average learning time for one image is around 160 steps, showing that greedy learning could cut ~25% off the expected training time (210 steps for one image). The overall testing accuracy on 10,000 unseen images from MNIST testing set reaches 78.9% and is 76.8 ± 0.8% on average, as illustrated in Figure 10, which is comparable with the float-precision baseline of 77.2% accuracy in Boybat et al. (2018).

In the next subsection, the immunity to RRAM device variations of so-trained SNNs is explored.

#### 3.2.5. Performance With Variations

The variations in RRAM crossbar arrays could be classified as two types: the cycle-to-cycle variation and the device-to-device variation. The cycle-to-cycle variation is mainly caused by the intrinsic stochastic physics mechanisms of the memristive devices. As mentioned in section 2.1, the conductance of our memristive devices is controlled by the states of the internal filaments. When a SET operation voltage is applied to the device, the oxygen vacancies will generate stochastically and vice versa for the RESET process. Therefore, the switching behaviors of memristive devices may vary from cycle to cycle, showing fluctuations even under the same operation conditions, which is known as the cycle-to-cycle variation. There also exists the device-to-device variation when it comes to RRAM arrays. The fabrication mismatches, line resistances, and capacitances will lead to different behaviors from device to device. For example, when pre-spikes/post-spikes are applied to one column/row of the array as illustrated in Figure 2, the actual voltage across each cell may vary due to the IR drop, and on the other hand, the threshold of each RRAM device is also different because of fabrication mismatches. Besides, the non-idealities of sources such as the misalignment for Gate pulses and SL pulses will also incur other variations during the training process, since the effective pulse width may vary in different operation cycles and for different cells. Proposing accurate physics and electronics models to predict the device manners is beyond the scope of this work (Yu et al., 2011a), so the impact of these variations on the proposed training methods is analyzed based on the variation of several main parameters on algorithm level, to evaluate the robustness of the proposed methods.

We have conducted repeated simulations with different levels of variation on the parameters: *A*_+_, *A*_−_, *W*_max_, *W*_min_, for both cycle-to-cycle (C2C) and device-to-device (D2D) variations. All variations are emulated by setting a certain level of the standard dispersion of the parameter, i.e., σ/μ (Querlioz et al., 2013; Agarwal et al., 2016; Gokmen and Vlasov, 2016). For D2D variation, the parameter will be sampled from the Gaussian distribution independently for all synapses before the start of one simulation, and this reference value for each synapse keep unchanged during the whole training process. If a C2C variation is also added to the simulation, the actual parameter for each synapse will be sampled from the Gaussian distribution regarding the D2D-varied value picked initially, every time the update operation happens.

The aim of the proposed greedy training method is to cooperate with the inevitable abrupt switching behavior existing in memristive devices, so the *A*_+_, *A*_−_ parameters are set to relatively large values (*A*_+_ = 1.0, *A*_−_ = 0.6 according to the experimental results in Figure 6), and the STDP learning window is as narrow as 4 timesteps to reduce the update operations on each synapse (update operations only happen when |Δ*t*| ≤ 2τ). Therefore a single update may cause a Δ*W* at the magnitude of 8 ~ 100% of the dynamic range, which indicates that 20-level devices could be sufficient for greedy training. Table 1 shows the impact of the *A*_+_, *A*_−_ variations. With both cycle-to-cycle and device-to-device variations, the accuracy drops from 76.8 to 73.9% at 30% variation level, which is already an extremely high level of variation for an electron device, but typical for research nanodevices (Querlioz et al., 2011). When the device-to-device *A*_+_, *A*_−_ variation reaches 50%, around 5% of devices could not be programmed properly in at least one direction (*A*_+_ or *A*_−_ becomes negative), i.e., the conductance of these defected devices always decreases whenever potentiation process happens and vice versa. In this situation, the accuracy drops around 10%. However, the functionality of the network is not challenged. On the other hand, the greedy training is immune to large cycle-to-cycle write variation up to 50%, since each device may suffer from a potentiation/depression disorder with a probability of only 5%, every time the update operation happens.

**Table 1:** The testing accuracy for different levels of variation on *A*_+_, *A*_−_.

**Variation level**	**10%**	**30%**	**50%**
C2C	76.22 ± 0.81%	75.30 ± 1.14%	74.84 ± 1.22%
D2D	76.42 ± 1.78%	76.17 ± 1.03%	65.42 ± 1.88%
Combined	75.74 ± 1.24%	73.94 ± 1.53%	63.48 ± 2.09%

*Results for cycle-to-cycle (C2C) variation, device-to-device (D2D) variation, and C2C-D2D combined variation are listed. Simulations are repeated for 12 times with each condition, and the testing accuracy is shown as μ±σ, where μ, σ represents the mean value and standard deviation respectively*.

We also simulated the impact of the dynamic range (*W*_max_, *W*_min_) variations, as shown in Table 2. The initial dynamic range is set to 10 ~ 50 μS, meaning that the on/off ratio equals to only 5, which is easy to fulfill for typical memristive devices (Kuzum et al., 2013). The network can tolerate 10% variation level of *W*_max_ and *W*_min_ with <2% accuracy loss, and still functions well with 30% cycle-to-cycle and device-to-device *W*_max_, *W*_min_ variation with a 67% testing accuracy. When the variation goes to 50%, around 10% of devices in the simulation are stuck at the initial value since the maximal conductance becomes less than minimal conductance, which incurs severe accuracy loss for MNIST application. Querlioz et al. (2011) have shown that this type of unsupervised SNN can tolerate 50% *W*_max_, *W*_min_ variation well, however with a dynamic range of 10^4^, which allows larger variations but is hard to implement for most nanodevices.

**Table 2:** The testing accuracy for different levels of variation on *W*_max_, *W*_min_.

**Variation level**	**10%**	**30%**	**50%**
C2C	76.67 ± 0.76%	71.90 ± 1.75%	63.94 ± 1.34%
D2D	74.91 ± 1.09%	71.60 ± 0.69%	65.20 ± 1.15%
Combined	75.34 ± 0.94%	67.20 ± 2.21%	56.16 ± 1.73%

*Results for cycle-to-cycle (C2C) variation, device-to-device (D2D) variation, and C2C-D2D combined variation are listed. Simulations are repeated for 12 times with each condition, and the testing accuracy is shown as μ±σ, where μ, σ represents the mean value and standard deviation respectively*.

Table 3 compares the performance between greedy-trained unsupervised SNNs and conventional-trained unsupervised SNNs (Querlioz et al., 2011; Boybat et al., 2018). The listed three networks are of the same structure, 784 inputs together with 50 output neurons. The learning increments and decrements (normalized by dynamic range) for greedy training and conventional training are compared, and we can see that conventional training requires the synapses to be able to tune their conductances at the magnitude of 0.5% to 1% regarding the switching window width (*W*_max_−*W*_min_), which needs devices to have over 200 levels under consecutive programming pulses (Querlioz et al., 2011). Since this requirement is hard to fulfill for most memristive devices (Gao et al., 2015; Park et al., 2016), an architecture wrapping N devices as one single synapse has been proposed by Boybat et al. (2018), and they have proved that training SNNs using up to 9 devices/synapse can achieve over 70% testing accuracy on MNIST, reducing the required device levels to around 20, which is easy to implement. On the other hand, the greedy training method proposed in this work dilutes the spiking activities in the time domain, and forces the synapses to learn greedily, with large learning increments and decrements of 30 to 50% regarding the switching window, therefore using one memristive device with 20 levels as one synapse could be sufficient to achieve the same functionality.

**Table 3:** Comparison table of memristive-device-based SNNs for MNIST handwritten recognition.

	**This work**	**Boybat et al., 2018**	**Querlioz et al., 2011**
Training method	Greedy	Conventional	Conventional
Network structure	784 × 50	784 × 50	784 × 50
Accuracy with variations	~75%	~70%	~80%
Devices per synapse	1	≥9	1
Learning increments, decrements	~0.5, ~0.3	0.01, 0.006	0.01, 0.005
Required device levels	~20	~20	>200

*The accuracy with variations of this work is obtained with 30% cycle-to-cycle and device-to-device A_+_, A_−_ variation, and 10% cycle-to-cyle and device-to-device W_max_, W_min_ variation. For Boybat et al. (2018), the N-in-1 architecture (non-differential) with N=9 and with device variation model is listed. And for Querlioz et al. (2011), the data is obtained with 25% cycle-to-cycle A_+_, A_−_ variation, and 25% cycle-to-cycle W_max_, W_min_ variation*.

## 4. Discussion

### 4.1. Device Endurance

**Figure 11:**
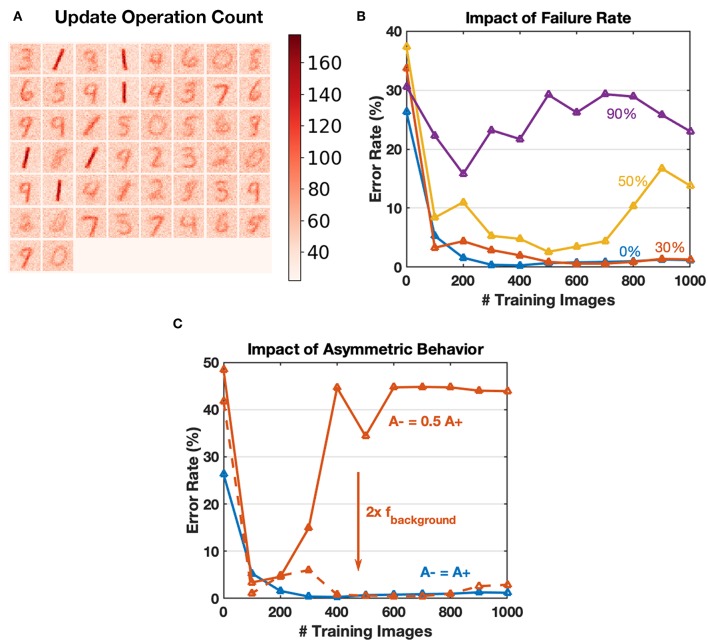
**(A)** Heat map of the update counts for each synapse after learning 60,000 MNIST training images. The maximum is <200, which could be ignored for endurance related problems. **(B)** Impact of array failure rate. A simple SNN of 4 output neurons to recognize 1,000 “0,” “1” images is simulated. The failed devices are kept at the initial state and do not respond to any input. The failure rate could have an impact on the convergence, and 30% failure rate can be tolerated in this application. **(C)** Using algorithm-level parameters to compensate for the common asymmetric switching behaviors for RRAM devices. For the solid red curve, double the background firing rate factor leads to similar performance with balanced switching conditions.

Online training for neural networks on RRAM devices often requires a large number of conductance tuning operations, where we must consider the device endurance problem. The core concept of greedy training is to dilute spike trains in the time domain, thus reducing the number of device operations. Typical update count map after training with 60,000 images is shown in Figure 11A, where update count of an individual synapse is no more than 200 times. The endurance related problems could be ignored for greedy training learning MNIST digits since these problems usually appear after 10^5^ operating pulses (Zhao et al., 2018). The parameters used by Boybat et al. (2018) indicate that the learning window for STDP lasts for over 200 timesteps, and at each time step, about ten spikes (calculated according to the MNIST statistics and firing rate mentioned) are generated at the input layer. The output layer is expected to have five spikes fired for each image as well. Therefore, an estimate of update operation number would be 200 × 10 × 5 = 10 k for one training image, while the value for the proposed greedy training is around 6 × 3 × 1 ≈ 20, reducing update operations by a factor of 500. The conventional training method may be affected by endurance related problems more severely. Besides, reducing the number of update operations could also make the algorithm more energy efficient theoretically.

### 4.2. Array Failure Rate

Although the endurance related device failure problem could be ignored for greedy training, we have conducted simple simulations to explore the influence of yield. A SNN with four output neurons is used to recognize 1,000 “0,” “1” digit images, and trained with different array failure rates. The failed devices are stuck to their initial states and do not respond to any input during training. Figure 11B shows that the convergence is affected severely, especially when the failure rate goes over 50%. Since endurance issues are ignored, a typical failure rate of a functional array should be around 10% (Wu et al., 2017), and greedy training is robust for this situation.

### 4.3. Compensate Asymmetric Switching Behavior

Commonly, memristive devices have asymmetric switching behaviors (Kuzum et al., 2013), which is one of the bottlenecks for hardware neural networks. Thanks to the pattern/background phases of greedy training, the potentiation and depression during SNN training happen in different time slots, and the input firing rate for each phase could be configured independently. Therefore, we can compensate for the asymmetric switching behavior partly by tuning the pattern/background firing factors, as shown in Figure 11C.

### 4.4. Divide Spikes Into Pattern/Background Parts

For greedy training, it is guaranteed that potentiation happens in the pattern phase and depression in the background phase. So we can divide the pre-spikes and post-spikes into minor parts from their timing middle points, then we get a negative/positive pulse pair for each spike (the same manipulation should be applied to gate-control signals as well). The original design of waveforms in Figure 3 requires post-spikes and gate-control signals to be synchronized well, so if the circuit non-idealities result in the misalignment of post-spikes and gate-control signals, there may cause unsafe device operations (V_G_^RESET^ applied to the gate node when SET is expected). Fortunately, breaking each spike signal into two parts and operates separately in the pattern and background phase could solve this problem. Jittering between G and SL signals will only lead to a lower effective overlapped pulse width, will not cause unsafe operations anymore.

## 5. Conclusion

To work with the inevitable large conductance change step introduced by RRAM devices, we propose novel approaches of pattern/background phases and greedy training for unsupervised SNNs. Pattern/background phases and greedy training method provide an efficient workflow of unsupervised SNN learning because they make sure that only the pattern spikes occur just before the post-spike events, and background spikes will follow the post-spikes. Furthermore, greedy training guarantees that only one post-spike will be fired for each stimulus, which allows larger weight changes. The simulated SNN model manages to cooperate with the large learning rate incurred by RRAM devices by diluting spikes in the temporal dimension and therefore achieves gradual learning with very few spikes, which significantly reduce the requirement on the number of gradual levels of memristive devices from over 200 to around 20, and then could be fulfilled by typical memristive devices. The greedy-trained unsupervised SNNs also have good immunity to the conductance change variation and switching window variation and reach ~75% testing accuracy on the MNIST test set with moderate variations. Furthermore, the low-density interaction fashion of greedy training reduces the number of SET/RESET operations on memristive devices by around 2 orders, for example a maximum of 200 operations is observed for single-epoch learning 60,000 MNIST training images, and this could substantially mitigate the endurance related problems which is one of the bottlenecks for memristive devices based online learning systems. This work shows the potential of RRAM devices serving as neuromorphic hardware to implement practical applications with properly-trained SNNs, even with various imperfect behaviors.

## Data Availability

The MNIST dataset used for this study can be found in THE MNIST DATABASE of handwritten digits.

## Author Contributions

The ideas and methods are proposed and discussed by YG, HW, and BG. The experiments and simulations mentioned in this work are completed by YG. During the whole progress, HW, BG, and HQ all offered suggestions which help YG to carry out the research reported by this article.

### Conflict of Interest Statement

The authors declare that the research was conducted in the absence of any commercial or financial relationships that could be construed as a potential conflict of interest.

## Footnotes

^1^The MNIST dataset used for this study can be found in THE MNIST DATABASE of handwritten digits.

## References

[B1] Agarwal S. Plimpton S. J. Hughart D. R. Hsia A. H. Richter I. Cox J. A. (2016). Resistive memory device requirements for a neural algorithm accelerator, in 2016 International Joint Conference on Neural Networks (IJCNN) (Vancouver, BC: IEEE), 929–938. 10.1109/IJCNN.2016.7727298

[B2] Ambrogio S. Balatti S. Milo V. Carboni R. Wang Z. Calderoni A. (2016). Novel rram-enabled 1t1r synapse capable of low-power stdp via burst-mode communication and real-time unsupervised machine learning, in 2016 IEEE Symposium on VLSI Technology (Honolulu, HI: IEEE), 1–2. 10.1109/VLSIT.2016.7573432

[B3] Ambrogio S. Balatti S. Nardi F. Facchinetti S. Ielmini D. (2013). Spike-timing dependent plasticity in a transistor-selected resistive switching memory. Nanotechnology 24:384012. 10.1088/0957-4484/24/38/384012 23999495

[B4] Bi G.-q. Poo M.-m. (1998). Synaptic modifications in cultured hippocampal neurons: dependence on spike timing, synaptic strength, and postsynaptic cell type. J. Neurosci. 18, 10464–10472. 9852584 10.1523/JNEUROSCI.18-24-10464.1998 PMC6793365

[B5] Boybat I. Le Gallo M. Nandakumar S. R. Moraitis T. Parnell T. Tuma T.. (2018). Neuromorphic computing with multi-memristive synapses. Nat. Commun. 9:2514. 10.1038/s41467-018-04933-y 29955057 PMC6023896

[B6] Carlson K. D. Richert M. Dutt N. Krichmar J. L. (2013). Biologically plausible models of homeostasis and stdp: stability and learning in spiking neural networks, in The 2013 International Joint Conference on Neural Networks (IJCNN) (Dallas, TX: IEEE), 1–8. 10.1109/IJCNN.2013.6706961

[B7] Chang C.-C. Liu J.-C. Shen Y.-L. Chou T. Chen P.-C. Wang I.-T. (2017). Challenges and opportunities toward online training acceleration using rram-based hardware neural network, in 2017 IEEE International Electron Devices Meeting (IEDM) (San Francisco, CA: IEEE), 11–6. 10.1109/IEDM.2017.8268373

[B8] Diehl P. U. Cook M. (2015). Unsupervised learning of digit recognition using spike-timing-dependent plasticity. Front. Comput. Neurosci. 9:99. 10.3389/fncom.2015.00099 26941637 PMC4522567

[B9] Eryilmaz S. B. Joshi S. Neftci E. Wan W. Cauwenberghs G. Wong H.-S. P. (2016). Neuromorphic architectures with electronic synapses, in 2016 17th International Symposium on Quality Electronic Design (ISQED) (Santa Clara, CA: IEEE), 118–123. 10.1109/ISQED.2016.7479186

[B10] Gao L. Wang I.-T. Chen P.-Y. Vrudhula S. Seo J.-s. Cao Y.. (2015). Fully parallel write/read in resistive synaptic array for accelerating on-chip learning. Nanotechnology 26:455204. 10.1088/0957-4484/26/45/455204 26491032

[B11] Gokmen T. Vlasov Y. (2016). Acceleration of deep neural network training with resistive cross-point devices: design considerations. Front. Neurosci. 10:333. 10.3389/fnins.2016.00333 27493624 PMC4954855

[B12] Guan X. Yu S. Wong H.-S. P. (2012). On the switching parameter variation of metal-oxide rrampart i: Physical modeling and simulation methodology. IEEE Trans. Elect. Dev. 59, 1172–1182. 10.1109/TED.2012.2184545

[B13] Jo S. H. Chang T. Ebong I. Bhadviya B. B. Mazumder P. Lu W. (2010). Nanoscale memristor device as synapse in neuromorphic systems. Nano Lett. 10, 1297–1301. 10.1021/nl904092h 20192230

[B14] Kistler W. M. Hemmen J. L. v. (2000). Modeling synaptic plasticity in conjunction with the timing of pre-and postsynaptic action potentials. Neural Comput. 12, 385–405. 10.1162/089976600300015844 10636948

[B15] Kuzum D. Jeyasingh R. G. Lee B. Wong H.-S. (2011). Nanoelectronic programmable synapses based on phase change materials for brain-inspired computing. Nano Lett. 12, 2179–2186. 10.1021/nl201040y 21668029

[B16] Kuzum D. Yu S. Wong H. S. (2013). Synaptic electronics: materials, devices and applications. Nanotechnology 24:382001. 10.1088/0957-4484/24/38/382001 23999572

[B17] LeCun Y. Boser B. Denker J. S. Henderson D. Howard R. E. Hubbard W. (1989). Backpropagation applied to handwritten zip code recognition. Neural Comput. 1, 541–551.

[B18] LeCun Y. Bottou L. Bengio Y. Haffner P. (1998). Gradient-based learning applied to document recognition. Proc. IEEE 86, 2278–2324.

[B19] Liu H. Lv H. Yang B. Xu X. Liu R. Liu Q. (2014). Uniformity improvement in 1t1r rram with gate voltage ramp programming. IEEE Elect. Dev. Lett. 35, 1224–1226. 10.1109/LED.2014.2364171

[B20] Maass W. (1997). Networks of spiking neurons: the third generation of neural network models. Neural Netw. 10, 1659–1671.

[B21] Masquelier T. Guyonneau R. Thorpe S. J. (2009). Competitive stdp-based spike pattern learning. Neural Comput. 21, 1259–1276. 10.1162/neco.2008.06-08-804 19718815

[B22] Masquelier T. Thorpe S. J. (2007). Unsupervised learning of visual features through spike timing dependent plasticity. PLoS Comput. Biol. 3:e31. 10.1371/journal.pcbi.0030031 17305422 PMC1797822

[B23] Morrison A. Diesmann M. Gerstner W. (2008). Phenomenological models of synaptic plasticity based on spike timing. Biol. Cybernet. 98, 459–478. 10.1007/s00422-008-0233-1 18491160 PMC2799003

[B24] Painkras E. Plana L. A. Garside J. Temple S. Galluppi F. Patterson C. (2013). Spinnaker: a 1-w 18-core system-on-chip for massively-parallel neural network simulation. IEEE J. Solid State Circ. 48, 1943–1953. 10.1109/JSSC.2013.2259038

[B25] Panda P. Allred J. M. Ramanathan S. Roy K. (2018). Asp: learning to forget with adaptive synaptic plasticity in spiking neural networks. IEEE J. Emerg. Select. Top. Circ. Syst. 8, 51–64. 10.1109/JETCAS.2017.2769684

[B26] Park J. Kwak M. Moon K. Woo J. Lee D. Hwang H. (2016). Tio x-based rram synapse with 64-levels of conductance and symmetric conductance change by adopting a hybrid pulse scheme for neuromorphic computing. IEEE Elect. Dev. Lett. 37, 1559–1562. 10.1109/LED.2016.2622716

[B27] Pedretti G. Milo V. Ambrogio S. Carboni R. Bianchi S. Calderoni A.. (2017). Memristive neural network for on-line learning and tracking with brain-inspired spike timing dependent plasticity. Sci. Rep. 7:5288. 10.1038/s41598-017-05480-0 28706303 PMC5509735

[B28] Prezioso M. Mahmoodi M. R. Bayat F. M. Nili H. Kim H. Vincent A.. (2018). Spike-timing-dependent plasticity learning of coincidence detection with passively integrated memristive circuits. Nat. Commun. 9:5311. 10.1038/s41467-018-07757-y 30552327 PMC6294012

[B29] Qiao N. Mostafa H. Corradi F. Osswald M. Stefanini F. Sumislawska D.. (2015). A reconfigurable on-line learning spiking neuromorphic processor comprising 256 neurons and 128k synapses. Front. Neurosci. 9:141. 10.3389/fnins.2015.00141 25972778 PMC4413675

[B30] Querlioz D. Bichler O. Dollfus P. Gamrat C. (2013). Immunity to device variations in a spiking neural network with memristive nanodevices. IEEE Trans. Nanotechn. 12, 288–295. 10.1109/TNANO.2013.2250995

[B31] Querlioz D. Bichler O. Gamrat C. (2011). Simulation of a memristor-based spiking neural network immune to device variations, in The 2011 International Joint Conference on Neural Networks (San Jose, CA: IEEE), 1775–1781. 10.1109/IJCNN.2011.6033439

[B32] Schemmel J. Briiderle D. Griibl A. Hock M. Meier K. Millner S. (2010). A wafer-scale neuromorphic hardware system for large-scale neural modeling, in Proceedings of 2010 IEEE international symposium on Circuits and systems (ISCAS) (Paris: IEEE), 1947–1950. 10.1109/ISCAS.2010.5536970

[B33] Wang Z. Ambrogio S. Balatti S. Ielmini D. (2015). A 2-transistor/1-resistor artificial synapse capable of communication and stochastic learning in neuromorphic systems. Front. Neurosci. 8:438. 10.3389/fnins.2014.00438 25642161 PMC4295533

[B34] Wu H. Yao P. Gao B. Wu W. Zhang Q. Zhang W. (2017). Device and circuit optimization of rram for neuromorphic computing, in 2017 IEEE International Electron Devices Meeting (IEDM) (San Francisco, CA: IEEE), 11–5. 10.1109/IEDM.2017.8268372

[B35] Wu M.-C. Lin Y.-W. Jang W.-Y. Lin C.-H. Tseng T.-Y. (2011). Low-power and highly reliable multilevel operation in zro2 1t1r rram. IEEE Elect. Dev. Lett. 32, 1026–1028. 10.1109/LED.2011.2157454

[B36] Wu X. Saxena V. (2017). Enabling bio-plausible multi-level stdp using cmos neurons with dendrites and bistable rrams, in 2017 International Joint Conference on Neural Networks (IJCNN) (Anchorage, AK: IEEE), 3522–3526. 10.1109/IJCNN.2017.7966299

[B37] Yao P. Wu H. Gao B. Eryilmaz S. B. Huang X. Zhang W.. (2017). Face classification using electronic synapses. Nat. Commun. 8:15199. 10.1038/ncomms15199 28497781 PMC5437298

[B38] Yao P. Wu H. Gao B. Zhang G. Qian H. (2015). The effect of variation on neuromorphic network based on 1t1r memristor array, in 2015 15th Non-Volatile Memory Technology Symposium (NVMTS) (Beijing: IEEE), 1–3. 10.1109/NVMTS.2015.7457492

[B39] Yu S. Gao B. Fang Z. Yu H. Kang J. Wong H.-S. (2013). A low energy oxide-based electronic synaptic device for neuromorphic visual systems with tolerance to device variation. Adv. Mater. 25, 1774–1779. 10.1002/adma.201203680 23355110

[B40] Yu S. Guan X. Wong H.-S. P. (2011a). On the stochastic nature of resistive switching in metal oxide rram: Physical modeling, monte carlo simulation, and experimental characterization, in 2011 International Electron Devices Meeting (Washington, DC: IEEE), 17–3. 10.1109/IEDM.2011.6131572

[B41] Yu S. Wu Y. Jeyasingh R. Kuzum D. Wong H.-S. P. (2011b). An electronic synapse device based on metal oxide resistive switching memory for neuromorphic computation. IEEE Trans. Elect. Dev. 58, 2729–2737. 10.1109/TED.2011.2147791

[B42] Zhao M. Wu H. Gao B. Sun X. Liu Y. Yao P. (2018). Characterizing endurance degradation of incremental switching in analog rram for neuromorphic systems. in 2018 IEEE International Electron Devices Meeting (IEDM) (San Francisco, CA: IEEE), 20–2. 10.1109/IEDM.2018.8614664

